# Synbiotics and Treatment of Asthma: A Double-Blinded, Randomized, Placebo-Controlled Clinical Trial

**DOI:** 10.31661/gmj.v8i0.1350

**Published:** 2019-06-28

**Authors:** Maryam Hassanzad, Keyvan Maleki Mostashari, Hosseinali Ghaffaripour, Habib Emami, Samane Rahimi Limouei, Ali Akbar Velayati

**Affiliations:** ^1^Pediatric Respiratory Diseases Research Center (PRDRC), National Research Institute of Tuberculosis and Lung Disease (NRITLD), Shahid Beheshti University of Medical Sciences, Tehran, Iran; ^2^Chronic Respiratory Diseases Research Center (PRDRC), National Research Institute of Tuberculosis and Lung Diseases (NRITLD), Shahid Beheshti University of Medical Sciences, Tehran, Iran; ^3^Shahid Beheshti University of Medical Sciences, Tehran, Iran; ^4^Mycobacteriology Research Center, National Research Institute of Tuberculosis and Lung Diseases (NRITLD), Shahid Beheshti University of Medical Sciences, Tehran, Iran

**Keywords:** Asthma, Synbiotics, Probiotics, Iran, Kidilact®

## Abstract

**Background:**

We examined the efficiency and safety of a specific synbiotic compound, brand name Kidilact^®^, in the treatment of asthma in children 12 years of age or younger.

**Materials and Methods:**

This double-blinded, randomized, placebo-controlled clinical trial was conducted in Tehran, Iran, from May 22, 2016, to May 21, 2017. One hundred children, 12 years of age or younger, who suffered from mild to moderate asthma were recruited in this study. The subjects were randomly divided into two groups; the experimental group received a sachet of Kidilact^®^, and the control group received a sachet of placebo once a day for six months. Both groups were compared in terms of the frequency of asthma attacks that were severe enough to require administration of fast-acting medications, the number of outpatient visits for asthma-related problems, and the frequency of hospitalization due to exacerbated symptoms of asthma.

**Results:**

There were fewer complaints of drug-induced side effects, e.g., vomiting, headache, stomachache, and diarrhea, exacerbated cough, and constipation in the experimental group than in the control group. Overall, a significantly greater number of participants in the experimental group were satisfied with the therapeutic intervention than those in the control group, as verified by the participants and their parents/guardians self-report. There was no significant difference between both groups in the frequency of asthma attacks and hospitalization due to exacerbated symptoms of asthma. The only significant difference between both groups was the count of outpatient visits. While the control group made 55 outpatient visits to the hospital, participants in the experimental group visited the hospital only 19 times (P=0.001).

**Conclusion:**

Results of our study indicates that synbiotic compound Kidilact^®^ generally alleviates the symptoms of asthma in children of 12 years of age or younger, resulting in less frequent outpatient visits to the hospital due to asthma-related problems while rarely causing any side effects. Due to ease of use, the rarity of side effects, and their indirect positive effects on quality of life of asthmatic patients, we recommend that synbiotics be incorporated in regular treatment and management of children with asthma.

## Introduction

Asthma is a chronic inflammatory condition of the lung airways that results in episodic airflow obstruction. It is one of the most common non-communicable diseases worldwide, and its prevalence is increasing [1-3]. In a review of 28 articles about pediatric asthma in Iran published between 1992 to 2012, which had a total of 96822 participants, the prevalence of asthma ever was reported to be 2.7% (95% confidence interval [CI]: 1.9 to 3.6) and 3.5% (95% CI: 2.6 to 4.6) in children of 6-7 and 13-14 years, respectively. The prevalence of wheezing in the past 12 months was reported to be 7.6% (95% CI: 5.6 to 9.8) and 10.7% (95% CI: 8.9 to 12.7) in children of 6-7 and 13-14 years, respectively. The prevalence of asthma has increased during the last two decades. Asthma creates a major burden on the health and economy of society due to mortality, morbidity, lost school days for children and lost workdays for parents. It also diminishes the quality of life of those who are affected by it and their families. A variety of primary risk factors contributes to continuous childhood asthma. Lower respiratory tract infections, pneumonia, severe bronchiolitis that requires hospitalization, and also exposure to tobacco smoke [4] are among these factors.

The digestive system provides the largest contact area between the body and the environment and is probably the most important battlefield for the immune system. Understanding the role of the digestive system, especially the guts, in regulating the function of the immune system, and recognizing probiotics and their therapeutic use in asthma-related diseases [5] have given scientists a renewed hope for the discovery of new medications for treatment of asthma.Probiotics are non-pathogenic organisms found in foods and can colonize when eaten. Probiotics positively affect the health of their host. They can also change the microbial ecology of the intestine [6, 7]. Researchers have shown that probiotics not only boost the function of our digestive system but also affect our body more systemically by changing the reaction of our immune system [8]. Probiotics are indigestible carbohydrates and are naturally sensitive to antibiotics. They are mostly from the oligosaccharides that reach the colon intact. They grow and increase the activity of commensal bacteria in the colon [9]. Not all probiotics work the same way. Probiotics also differ in terms of their level of safety and efficiency. For example, bifidobacteria are non-pathogenic friendly bacteria uniquely suitable for infants and children. The major part of the intestinal flora of breastfeeding infants consists of bifidobacteria. Powdered milk prebiotics are different from breast milk prebiotics (human milk oligosaccharides) [10, 11]. Short-chain prebiotics (galactooligosaccharides) and long-chain prebiotics (e.g., fructooligosaccharides) in a dose-dependent way increase the growth of Bifidobacteria [12]. The resulted microbial flora is similar to the microbial flora of the breastfeeding infants [13]. Because the lack of asthma control in children is problematic and can lead to attacks that could endanger the patient’s life, there is a need to prevent the risk of asthma and enhance the level of immunity. Synbiotics are composed of prebiotics and probiotics and it companionship guarantees the survival of prebiotics and increases the rate of their colonization and growth [14, 15]. Prebiotics can be effective in diminishing the prevalence of asthma by reducing the incidence of atopic dermatitis, lower respiratory tract infections, pneumonia, bronchiolitis, allergic rhinitis, food allergies, and allergy to aeroallergens. The objective of the present survey was to examine the efficiency and safety of a specific synbiotic compound, brand name Kidilact®, in the treatment of asthma in children of 12 years of age or younger.

## Materials and Methods

### Trial Design

This study was a double-blind, randomized, placebo-controlled clinical trial conducted between May 22nd, 2016 and May 21st, 2017.

### Participants

Participants were recruited from among children, 12 years of age or younger, who visited the pediatric pulmonology clinic of Masih Daneshvari Hospital for treatment of mild to moderate asthma between May 22, 2016, and November 21, 2017. The severity of asthma was determined using the guidelines provided by the Global Initiative for Asthma (GINA) [16]. The exclusion criteria were other accompanying diseases, inability to commit to timely and regular medication consumption, severe side effects, and being under any other type of treatment that might affect the study results. Any complications caused by the administration of probiotics to patients, such as gastrointestinal complications or intolerance, led to their exclusion from the study. Before the study began, one of the researchers explained the study protocol to the parents/guardians of the children. The objective of the study was also explained to the participants’ parents/guardians. The parents/guardians were also told that they could withdraw their children from participation in the study at any time without being asked for a reason. They were also assured that withdrawal from the study would not have any detrimental effect on their relationship with the medical personnel and would not affect the quality of the medical services their children received. Participants and their parents/guardians were assured that the collected information would be kept confidential. Adequate measures were taken to protect the privacy of participants and to maintain the confidentiality of the data. Participants were assigned a number ID, and this number was used during data collection and processing. One hundred parents/guardians volunteered their children to participate in this study. All parents/guardians provided permission for their children’s participation in the study by signing a consent form. The duration of the trial for each participant was six months. The study was completed on May 21, 2017. All environmental conditions were similar in both groups.

### Interventions

Participants were randomly divided into two groups; the experimental group (n=51) and control group (n=49). Random allocation of participants to either the experimental or control group was done by a coin toss. Twelve packages of medication (or placebo) were considered for each participant. Participants were given four packages of medication at the beginning of the study, and the next eight packages were given to them on the two follow-up sessions (4 packages on each session). Each package included 15 sachets of placebo or Kidilact®. Parents/guardians were instructed to keep the packages refrigerated, and each day dissolve the contents of one sachet in water and feed it to their child. They were also advised that the medication should not be taken with hot food or beverages. Parents were offered training on how to use the medication; however, the procedure was very simple, and none of the parents/guardians asked for training. The participants’ safety was of upmost importance to us. Prebiotics and probiotics are frequently used in the food industry. Their use has been approved by both Iran and the USA Food and Drug Administration (FDA) organization. Nevertheless, participants always had access to the medical center, and the staff was available to help in an unlikely event that an issue might arise. The frequency of asthma attacks that required fast-acting medications, the number of outpatient visits for asthma-related problems, and the frequency of hospitalization due to exacerbated symptoms of asthma were monitored and recorded using questionnaires, face-to-face meetings, and telephone follow-ups. Parents/guardians were also asked to monitor and record any side effects of the medications. During the study, participants were living in their homes and continuing with their usual activities of daily living. Parents/guardians played a key role in relaying relevant information to the researchers. For each participant, the clinical trial lasted for six months. During this period, the participant was scheduled to visit the hospital and meet with one of the researchers once every eight weeks. During these visits, the information was gathered from the participants. In every visit, a questionnaire including questions regarding the frequency of asthma attacks that required fast-acting medications, the number of outpatient visits for asthma-related problems, the frequency of hospitalization due to exacerbated symptoms of asthma, side effects, and also participants’ satisfaction with the medication (satisfied or unsatisfied) was used by the experimenter to ensure accurate recording of information. Participants’ and their parents’/guardian’s questions, if any, were also answered during these meetings.

### Specifications of the Synbiotic Compound Used In This Study

A synbiotic compound, a collection of high amounts of seven well-known bacterial probiotic strains (including the children’s specific strains, *Bifidobacterium infantis*, which is associated with fructooligosaccharide prebiotic and helps with the growth and function of probiotics), was used in this study. The compound’s brand name is Kidilact® and ismanufactured by Zist Takhmir Co., a knowledge-based company in Iran. Table-1 shows the composition of Kidilact®. The placebo used in this study was also manufactured by Zist Takhmir Company. The packaging and appearance of the placebo were exactly the same as Kidilact®. The only difference was that the placebo did not contain any pre- or probiotics. Zist Takhmir Company coded packages of placebo and Kidilact®. At the beginning of the trial, we ensured that participants in each group were given packages of the medication with the same code number (i.e., either the placebo or active medication). Only upon completion of the trial, the codes were revealed to us, and we could determine, which group had received placebo and which one had received Kidilact®. This was a double-blinded study; hence, neither the researchers nor the participants and their parents/guardians were aware of the nature of their medication, i.e., placebo vs. Kidilact® during the study.

### Outcomes

The primary outcomes of this study were the frequency of asthma attacks that required fast-acting medications, visits to a hospital due to respiratory complaints or exacerbated symptoms of asthma, and the rate of hospitalization due to asthma attacks. The secondary outcomes of the study were participants’ level of satisfaction from their treatment and the side effects of the medications. All patients receiving probiotics orally declared their satisfaction. These outcomes were compared between the experimental and control groups.

### Research Ethics

All of the patients received routine therapies in this study according to available clinical guidelines, and no changes were made to routine treatments. In the experimental group, in addition to the usual treatment, probiotics were also prescribed. The present study was approved by the research ethics committee of Shahid Beheshti University of Medical Sciences (IR.SBMU.NRITLD.REC.1394.217), and written consent forms filled out and signed by guardians/parents of all participants before starting the survey. Also, it was registered at Clinical Trial Registration Identifier (code: TCTR20180625002, www.clinicaltrials.in.th)

### Statistical Analysis

The SPSS software version 16 (SPSS Inc., Chicago, Ill., USA) was used for the statistical analysis. Qualitative data were examined using the Chi-square test. The spread or distribution of the data was tested with Fisher’s Exact Test. Quantitative variables were examined by t-test or its non-parametric equivalent, i.e., Mann-Whitney test. Kolmogorov-Smirnov test or Shapiro-Wilk test were used to examine the normality of data distribution. A P-value of less than 0.05 was considered significant. Quantitative data were described as mean and standard deviation while qualitative data were described as percentages and prevalence.

## Results

**Figure 1:**
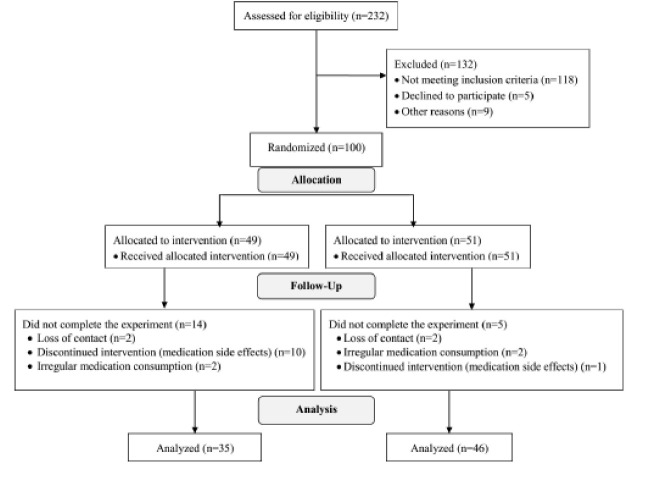
CONSORT diagram of the study

Overall, 19 participants, 5 from the experimental group and 14 from the control group, withdrew from the study (Figure-1). Therefore, data from 46 participants in the experimental group (29 males and 17 females) and 35 participants in the control group (19 male and 16 female) were available and used for analysis. Out of five participants in the experimental group who withdrew from the study, only one noted vomiting as the side effect that has discouraged him from participation. The other four either could not commit to the protocol, i.e., regular medication consumption, or were not interested in participating anymore, or had moved to another city and hence were not able to complete their participation in the study. Interestingly, a larger number of participants in the control group withdrew from the study due to the side effects. Out of 14 participants in the control group who quit the study, ten said they did so because of the medication’s side effects. The reported side effects varied and included vomiting, headache, stomachache and diarrhea, exacerbated cough, and severe constipation. Participants’ demographic information is summarized in Table-2. There was no significant difference between both groups in the term of participants’ age (P=0.718). The mean age of participants in the experimental and control group was 6.9 ±2.7 years and 6.6±2.4 years, respectively. The youngest participant was a 13-month-old baby boy, and the oldest one was a 12-year-old boy. Since one of the main signs of asthma control is the reduced need for fast-acting medications used during asthma attacks, we asked the participants and their parents/guardians to track how often the participants required fast-acting medications during the study. Furthermore, both the participants and their parents/guardians were asked to monitor and record the incidences of breathing difficulty and their severity, severe wheezing, and other signs of the asthma attack. Reviewing these records, we found that in general participants used fast-acting medications less frequently than expected. Four participants from each group required fast-acting medications during the study. There was a small difference in the percentage of participants in the two groups in terms of their need for fast-acting medication. Nine percent of individuals in the experimental group and 11% of those in the control group had used fast-acting medications during the study. Mann-Whitney test revealed that the difference was not significant (P=0.143). We also monitored and recorded participants’ visits to the hospital due to respiratory complaints or exacerbated symptoms of asthma. Since viruses are the most important causes of exacerbation of asthma symptoms, visits to the hospital due to common cold were also considered in this analysis. Results showed that while the control group had 55 outpatient visits to the hospital, the experimental group had visited the hospital only 19 times. Mann Whitney test revealed that the difference between the two groups in the number of visits to the hospital was significant (P=0.001). The number of hospital admission days (due to asthma attacks) was also tracked for each participant. Results showed that the incidence of hospitalization in the control group was almost four times higher in the control group than in the experimental group. In total, participants in the control group had a record of 11 days of admission to the hospital, while those in the experimental group had been hospitalized for only three days. However, the results of the Mann Whitney test revealed that the difference between both groups considering the days of hospitalization was not significant (P=0.453).To minimize any potential error, we combined the aforementioned three variables, i.e., need for fast-acting medications, visit to the hospital, and admission to the hospital, and considered them as a single indicator of the asthma attack. We called this indicator need for medical attention. Participants, who required at least one form of medical attention during the study, were labeled “Yes,” and those who did not require any form of medical attention were labeled “No.” Only seven of the 46 participants in the experimental group (15.2%) required some form of medical attention at least once. The other 39 participants in this group (84.8%) did not require any form of medical care during the study. In contrast, ten of the 35 participants in the control group (28.6%) required some form of medical attention at least once. The other 25 participants in this group (71.4%) did not require any form of medical care during the survey. Despite the relatively large difference between the two groups in the percentage of the participants who required a form of medical care, Chi-square analysis revealed that the difference was not significant (P=0.714), i.e., both groups were not significantly different in their need for medical care. We also asked the parents/guardians whether they were happy and satisfied with the effect of the medication or not. This was a ‘Yes or No’ question, i.e., if they were generally happy with the medical intervention, they would answer ‘Yes’ to this question. However, if they were generally unhappy with their children’s treatment, then their answer would be ‘No.’ Parents/guardians were asked to consider the ease of use and positive effects of medication, and also the existence of side effects when answering this question. Results showed that the subjects in the experimental group were significantly happier, i.e., they were more satisfied, with their treatment than subjects in the control group. When asked about their experience with medication, 78.3% of participants in the experimental group said they were happy and satisfied with their medication, while only 25.7% of those in the control group expressed satisfaction with their medication, i.e., placebo.

## Discussion

In this study, we examined the efficiency and safety of a specific synbiotic compound, i.e., Kidilact®, in the treatment of asthma in children 12 years old and younger. There were no previous studies performed in Iran that could provide us with a guideline on what type of synbiotic compound to use; therefore, we chose Kidilact® since it was the only product readily available in Iran at the time of the study and had the highest potential for effectiveness. We found that in general Kidilact® alleviates the symptoms of asthma in children of 12 years old or younger, while rarely causing any side effect. The experimental group who received Kidilact® required fast-acting medications less often, visited the hospital due to respiratory complaints or exacerbated symptoms of asthma less frequently, and were hospitalized for fewer days than those who received placebo. While the difference between the two groups in the number of visits to the hospital was significant, their differences in need for fast-acting medications and the number of days of hospitalization were not significant. Kidilact® contains *Lactobacillus acidophilus*, which might be effective in reducing the count and severity of respiratory infections among children [16]. Respiratory infections are probably the most common cause of exacerbation of symptoms of asthma. Therefore, it is possible by reducing the frequency and severity of respiratory infections; synbiotics have contributed to the reduced frequency of hospital visits among the experimental group. It should be noted that less frequent infections also translates to the less frequent use of antibiotics, and hence reduces the detrimental effect of antibiotics on intestinal microbial flora and the probiotics within the digestive system [17]. Previous researches on the effect of synbiotics on asthma have had conflicting results. For example, Van der AA *et al*. [18] showed that synbiotic mixtures could prevent asthma-like symptoms in infants with atopic dermatitis. In a double-blind, placebo-controlled trial they examined the effect of early intervention with synbiotics on the prevalence of asthma-like symptoms in ninety infants of 7 months old or younger, with atopic dermatitis. The results showed that the prevalences of frequent wheezing and wheezing and/or noisy breathing apart from colds were significantly lower in the infants who received synbiotic than in the control group. Furthermore, significantly fewer children in the synbiotic group than in the control group (5.6% vs. 25.6%) ended up using asthma medication. Chen *et al*. [19] also revealed that probiotic supplements might benefit school-age children suffering from allergic airway diseases such as asthma and allergic rhinitis. In a randomized, double-blind, placebo-controlled study, Chen *et al*. examined the effect of daily supplementation of a specific *L. gasseri* A5 for eight weeks on the clinical symptoms and immunoregulatory changes in children of 6-12 years old who suffered from asthma and allergic rhinitis. They found that the pulmonary function and peak expiratory flow rates were increased significantly, and the clinical symptoms of asthma and allergic rhinitis were decreased in the probiotic-treated children as compared to the control group. In contrast, Rose *et al*. [20] showed that *L. rhamnosus* (LGG), one of the most widely used probiotic strains, had no clinical effect on atopic dermatitis or asthma-related events (e.g., need of inhalation, symptom-free days), and only mild effects on allergic sensitization. In their double-blind study, Rose *et al*. examined the effect of LGG on 131 children (6-24 months old) with at least two wheezing episodes and a first-degree family history of atopic disease. The study took one year; six months intervention and six months’ follow-up. Their results showed that although the supplementation was well-tolerated by the children and no severe adverse event occurred; there was no significant difference between the experimental and control group in atopic dermatitis or asthma-related events. Niers *et al*. [21] examined the short- and long-term effects of intervention with probiotics on allergic airway disease. In their initial study, the effect of administration of a probiotic mixture (Ecologic®Panda) during pregnancy and the first year of life was examined in a randomized placebo-controlled trial. The study included 123 high-risk infants, i.e., infants with a positive family history of allergic diseases, such as atopic eczema, food allergy, asthma or allergic rhinitis. Their results showed that Ecologic®Panda reduced the incidence of eczema, but not atopic eczema. Furthermore, no difference was found in respiratory symptoms indicative of asthma or allergic rhinitis between both groups of infants once they had reached the age of 2 years. Parents of 83 infants participating in the initial study were willing to enroll their children in a follow-up study once they had reached the age of six years. During their long-term follow-up of a randomized placebo-controlled trial, Gorissen *et al*. [22] found no beneficial effect of prenatal and 1-year long post-natal use of Ecologic®Panda on the development of allergic diseases at the age of 6 years. Furthermore, the two groups were not significantly different in terms of the prevalence of asthma, allergic rhinitis, or food allergy. Besides, the positive effect of Ecologic®Panda on the prevalence of eczema, which was observed at the age of 2 years, was not present once the children had reached the age of 6 years. Based on the findings of these two studies, the authors concluded that administration of a selected combination of probiotics, i.e., Ecologic®Panda, during pregnancy and the first year of life had the beneficial effect on the development of eczema up to the age of 2 years. However, the beneficial effect did not extend to the age of 6 years and did not prevent asthma. The difference findings of these studies can be attributed to the differences in the participants’ age range, duration of medical intervention, and the type of probiotics and synbiotics used in each study. We asked the parents/guardians whether they were generally satisfied with the therapeutic intervention or not. They were also asked to consider the ease of use and positive effects of medication, and the existence of side effects when answering this question. We found that a significantly greater number of participants in the experimental group were satisfied with their treatment. The percentage of participants who were satisfied with their treatment was 78.3% and 25.7% for the experimental and control group, respectively. As we expected, Kidilact® was well accepted by the participants; only one child from the experimental group complained of vomiting as a side effect of the medication. The rarity of the side effects of probiotic has been previously reported too [22]. Williams [23] noted a temporary increase in gas and bloating as the most common reaction to bacteria-based probiotic supplements. While only one participant from the experimental group quit the study due to the side effects of medication, ten participants from the control group left the study due to the side effects. Since the only difference between Kidilact® and the placebo was that, the latter did not contain any pre- or probiotics; this unexpected finding requires further examination. The high level of satisfaction of physicians and patients with the probiotic treatment, and also the significant reduction in the number of outpatient visits and respiratory infections suggest that although probiotics can theoretically change the performance of the immune system from Th2 to Th1, most of their positive effect in asthma seems to occur by limiting infections and their side effects. By limiting pathological inflammation and converting them to physiological inflammation (in the intestines and possibly other parts of the body), probiotics restrict the dispersion of inflammatory agents.Also, by removing most of the factors that contribute to respiratory infections, the most important trigger of asthma attacks will be eliminated.The authors believe that participants’ satisfaction with the therapeutic approach used in the present study indicates that synbiotic compounds improve the patient’s quality of life.The impact of probiotics was real and tangible and manifested by reduced respiratory infections and hospital visits. Due to the nature of the study, we relied on participants and their parents/guardians in recording some of the information. Although participants and their parents/guardians had been given proper training as to how to identify and record such information (e.g., monitoring and recording the incidences of breathing difficulty and wheezing, and their severity), there is a chance that on occasions these symptoms have not been identified or recorded properly and hence have introduced error into the data. Furthermore, although originally we had a relatively large sample size, due to a rather large number of withdrawals the sample size shrank considerably. Future studies should aim at exploring the effect of synbiotics on treatment and control of asthma using a larger sample size. A larger sample may also allow examining the effects of synbiotics on treatment and control of asthma in males and females separately.

## Conclusion

Patients suffering from asthma and cause minimal side effects very well accept Synbiotics, specifically Kidilact®. Furthermore, they are easy to use and have acceptable stability under normal conditions. Due to their ease of use, limited side effects, and positive effects on patients’ quality of life, we recommend that synbiotics be considered and utilized in the management and treatment of asthma. Further research is required to determine the most effective synbiotic compounds for the treatment of asthma, the best time to use them and the optimal length of such therapeutic interventions.

## Acknowledgment

The authors would like to express their gratitude to the staff members of Zist Takhmir Co. for providing us with the medications used in this study. We also would like to thank Ms. Alizadeh for her invaluable help with the statistical analysis of the data. Research deputy of National Research Institute of Tuberculosis and Lung Disease (NRITLD) of Shahid Beheshti University of Medical Sciences supported this project (grant No.: 8116).

## Conflict of Interest

There is no conflict of interest to be declared.

**Table 1:** The Composition of Kidilact®, the Active Medicine Used in Our Study

*Lactobacillus Casei*	*Bifidobacterium infantis*
*Lactobacillus acidophilus*	*Bifidobacterium breve*
*Lactobacillus rhamnosus*	*Streptococcus thermophiles*
*Lactobacillus bulgaris*	Fructooligosaccharide (FOS)/[Prebiotic]

**Table 2:** Participant’s Demographic Information

**Variables**	**Experimental group** **(n=46)**	**Control group** **(n=35)**	**P-value**
Age, y*	6.9±2.7	6.6 ±2.4	0.718
**Sex**			
**Male**	29 (35.8%)	19 (23.5%)	0.497
**Female**	17 (21%)	16 (19.8%)	

*Data presented as mean±SD, ** Data presented as n (%)

## References

[1] Ghaffari J, Aarabi M (2013). The prevalence of pediatric asthma in the Islamic Republic of Iran: A systematic review and meta-analysis. J Pediatr Rev.

[2] Sennhauser FH, Braun-Fahrländer C, Wildhaber JH (2005). The burden of asthma in children: a European perspective. PaediatrRespir Rev.

[3] Kendig EL, Wilmott RW, Chernick V: Kendig and Chernick's disorders of the respiratory tract in children. Elsevier Health Sciences; 2012.

[4] Hassanzad M, Khalilzadeh S, Nobari SE, Bloursaz M, Sharifi H, Mohajerani SA, Nejad ST, Velayati AA (2015). Cotinine level is associated with asthma severity in passive smoker children. Iran J Allergy Asthma Immunol.

[5] Kalliomäki M, Salminen S, Arvilommi H, Kero P, Koskinen P, Isolauri E (2001). Probiotics in primary prevention of atopic disease: a randomised placebo-controlled trial. Lancet.

[6] Binns N: Internationsl life sciences institute (ISLI) Europe: concise monograph series. Probiotics, prebiotics and the gut microbiota. In.; 2013.

[7] Hill C, Guarner F, Reid G, Gibson GR, Merenstein DJ, Pot B, Morelli L, Canani RB, Flint HJ, Salminen S (2014). The International Scientific Association for Probiotics and Prebiotics consensus statement on the scope and appropriate use of the term probiotic. Nat Rev GastroenterolHepatol.

[8] Isolauri E, Sütas Y, Kankaanpää P, Arvilommi H, Salminen S (2001). Probiotics: effects on immunity. The American journal of clinical nutrition.

[9] Fouhy F, Guinane CM, Hussey S, Wall R, Ryan CA, Dempsey EM, Murphy B, Ross RP, Fitzgerald GF, Stanton C (2012). High-throughput sequencing reveals the incomplete, short-term recovery of infant gut microbiota following parenteral antibiotic treatment with ampicillin and gentamicin. Antimicrob Agents Chemother.

[10] Rougé C, Goldenberg O, Ferraris L, Berger B, Rochat F, Legrand A, Göbel UB, Vodovar M, Voyer M, Rozé J-C (2010). Investigation of the intestinal microbiota in preterm infants using different methods. Anaerobe.

[11] Boehm G, Stahl B (2007). Oligosaccharides from milk. J Nutr.

[12] Nauta AJ, Garssen J (2013). Evidence-based benefits of specific mixtures of non-digestible oligosaccharides on the immune system. CarbohydrPolym.

[13] Knol J, Scholtens P, Kafka C, Steenbakkers J, Gro S, Helm K, Klarczyk M, Schöpfer H, Böckler H-M, Wells J (2005). Colon microflora in infants fed formula with galacto-and fructo-oligosaccharides: more like breast-fed infants. J Pediatr Gastroenterology Nutr.

[14] Guinane CM, Cotter PD (2013). Role of the gut microbiota in health and chronic gastrointestinal disease: understanding a hidden metabolic organ. TherapAdvGastroenterol.

[15] Scholtens PA, Oozeer R, Martin R, Amor KB, Knol J (2012). The early settlers: intestinal microbiology in early life. Ann Rev Food Sci Technol.

[16] Masoli M, Fabian d, Holt S, Beasley R (2004). Global initiative for Asthma (GinA) program: the global burden of asthma: executive summary of the GinA dissemination committee report. Allergy.

[17] Aureli P, Capurso L, Castellazzi AM, Clerici M, Giovannini M, Morelli L, Poli A, Pregliasco F, Salvini F, Zuccotti GV (2011). Probiotics and health: an evidence-based review. Pharmacological research.

[18] Van der Aa L, Van Aalderen W, Heymans H, HenkSillevisSmitt J, Nauta A, Knippels L, Ben Amor K, Sprikkelman A, Group SS (2011). Synbiotics prevent asthma-like symptoms in infants with atopic dermatitis. Allergy.

[19] Chen YS, Lin YL, Jan RL, Chen HH, Wang JY (2010). Randomized placebo-controlled trial of lactobacillus on asthmatic children with allergic rhinitis. PediatrPulmonol.

[20] Rose M, Stieglitz F, Köksal A, Schubert R, Schulze J, Zielen S (2010). Efficacy of probiotic Lactobacillus GG on allergic sensitization and asthma in infants at risk. ClinExp Allergy.

[21] Niers L, Martín R, Rijkers G, Sengers F, Timmerman H, Van Uden N, Smidt H, Kimpen J, Hoekstra M (2009). The effects of selected probiotic strains on the development of eczema (the PandA study). Allergy.

[22] Gorissen D, Rutten N, Oostermeijer C, Niers L, Hoekstra M, Rijkers G, Van der Ent C (2014). Preventive effects of selected probiotic strains on the development of asthma and allergic rhinitis in childhood The Panda study. ClinExp Allergy.

[23] Williams NT (2010). Probiotics. American Journal of Health-System Pharmacy.

